# Supplementary material for: Ingroup sources enhance associative inference
Source: Commun Psychol. 2023 Dec 14;1:40. doi: 10.1038/s44271-023-00043-8 (PMC11332085; doi:10.1038/s44271-023-00043-8)
Supplement: Supplementary file 1 — Supplemental Material [file 44271_2023_43_MOESM1_ESM.pdf]

Supplementary Information

Supplementary Note 1: The group manipulation successfully created ingroup biases in liking and subjective encoding.

Two measures served for testing the success of the group manipulation: ingroup/outgroup liking and ease of encoding. First, we tested whether participants liked the ingroup personas more than the outgroup personas. Studies 1 and 2 used a 7-point relative liking scale, in which the outgroup persona formed the lower pole and the ingroup persona the higher pole. Each item (from the IL-6 [1]) was judged by which persona it applied to more. Responses were coded between -3 and +3, where values lower than zero and higher than zero indicated preferences for outgroup and ingroup, respectively. Supplementary Table 1 displays the liking values and shows strong ingroup favoritism before and after the memory task in both studies. While relative ingroup preference decreased during the experiment, it remained significantly above zero, with large effect sizes.

Supplementary Table 1.1

Relative Ingroup Liking in Studies 1 and 2

		Descriptives		*t*-test against 0				
	Time	*M*	*SD*	*df*	*t*	*p*	*d*	95%CI
1	pre	2.19	0.59	52	27.00	<.001	3.71	2.80, 4.62
	post	1.72	0.93	52	13.50	<.001	1.85	1.20, 2.51
2	pre	2.26	0.62	67	29.93	<.001	3.63	2.84, 4.42
	post	1.84	0.83	67	18.33	<.001	2.22	1.61, 2.84

Note. Means (*M*) and standard deviations (*SD*) for liking before (pre) and after (post) the memory task for Studies 1 and 2. Average mean was contrasted against zero in one-sample *t*-tests. The table shows the degrees of freedom (*df*), t-values (*t*), significance (*p*), and effect size (*d*) with a 95% Confidence Interval for each comparison.

In Study 3, participants judged liking for ingroup and outgroup separately on absolute scales from 1-7, where higher values indicated greater liking. This allowed us to test whether the ingroup was liked and the outgroup was disliked in absolute terms instead of a mere relative preference. Supplementary Table 1.2 shows that ingroup ratings were significantly higher than outgroup ratings. In addition to this comparison, we also contrasted each liking score against the midpoint of 4 and found that ingroup values were significantly above (pre: $t(67)=23.04$, $p<.001$, $d=2.79$, 95%CI[2.11, 3.47]; post: $t(67)=16.09$, $p<.001$, $d=1.95$, 95%CI[1.36, 2.54]), while outgroup values were significantly below that midpoint (pre: $t(67)=-9.39$, $p<.001$, $d=-1.14$, 95%CI[-1.66, -0.62]; post: $t(67)=-6.00$, $p<.001$, $d=-0.73$, 95%CI[-1.23, -0.23]).

Supplementary Table 1.2

Absolute Ingroup and Outgroup Liking in Study 3

	Ingroup		Outgroup		Paired *t*-test				
Time	*M*	*SD*	*M*	*SD*	*df*	*t*	*p*	*d*	95%CI
pre	5.91	0.69	2.75	1.10	67	18.21	<.001	2.21	1.76, 2.65
post	5.67	0.85	3.10	1.24	67	13.18	<.001	1.60	1.24, 1.96

Note. Means (*M*) and standard deviations (*SD*) for liking before (pre) and after (post) the memory task for Study 3. Average means in ingroup and outgroup were contrasted in paired *t*-tests.

Second, we looked at the ease of encoding ratings, which participants provided after each encoding display. We expected participants to rate the material presented by the ingroup as easier to encode than the material presented by the outgroup. This would corroborate the success of the group manipulation as the counterbalancing of material secured that group differences could not arise based on the stimuli themselves. Participants were asked to rate how easy memorizing the material they saw was. The scale ranged from 1 = easy to 3 = hard to encode. For this analysis, only the AB episodes,

which were directly paired with a persona face, were considered. Supplementary Table 1.3 illustrates that ingroup displays were consistently and significantly rated as easier to encode than outgroup displays.

Supplementary Table 1.3

Rated Ease of Encoding in Ingroup and Outgroup Displays

	Ingroup		Outgroup		Paired *t*-test				
	M	*SD*	*M*	*SD*	*df*	*t*	*p*	*d*	95%CI
1	2.05	0.40	2.22	0.38	52	6.11	<.001	0.84	0.52, 1.16
2	1.96	0.42	2.12	0.42	67	5.16	<.001	0.63	0.36, 0.89
3	1.94	0.42	2.08	0.34	67	4.41	<.001	0.53	0.28, 0.79

Note. Means and Standard Deviations in all Studies. Ingroup and outgroup values were compared in paired *t*-tests.

Supplementary Note 2: Ingroup source memory is lower when encoding is difficult.

To further investigate the source memory results, we tested whether source memory was responsive to ease of encoding ratings in trials with correct inference. We expected that for AC correct inferences ingroup source memory would be reduced when participants found encoding challenging, while outgroup source memory would not be responsive to encoding difficulty (see Study 1 Results).

We summed the subjective ease of encoding for each triplet from AB and BC trials. Based on each participant's rating distribution, the resulting ratings were split into three categories (easy, middle, hard to encode). This approach allowed us to account for individual biases in ease of encoding ratings, where some participants may have been drawn to one or the other end of the scale. Using these ratings, ANOVAs for team memory were conducted with the factors of group (ingroup, outgroup) and ease of encoding categories (easy, middle, hard). To assess whether the emerging pattern was similar to the source memory pattern, *t*-tests were carried out as direct contrasts. The analysis was only conducted with memory for the team, as persona memory did not show the pattern of lowered ingroup source memory when inference was incorrect.

In Study 1, nine participants were excluded from the analysis for not having sufficient variance in the encoding ratings for ingroup and outgroup. On the ANOVA, there was an effect of ease of encoding (F(2,86)=3.70, p=.029, η^2=.01), reflecting that there was better source memory for episodes judged easier to encode. There were no effects of group (F(1,43)=0.73, p=.397, η^2=.003) and no interaction (F(2,86)=1.78, p=.174, η^2=.01). Nevertheless, direct contrasts showed that in the ingroup, source memory for team was better in easy trials compared to medium (t(43)=2.10, p=.042, d=0.32, 95%CI[0.01, 0.62]) and hard trials (t(43)=3.03, p=.004, d=0.46, 95%CI[0.14, 0.77]) with no difference between medium and hard trials (t(43)=0.91, p=.368, d=0.14, 95%CI[-0.16, 0.44]). In contrast, outgroup persona memory was similar across encoding ratings (easy vs medium: t(43)=0.16, p=.871, d=0.02, 95%CI[-0.28, 0.32], BF_{01}=6.05, $Md_{posterior}$=0.02, $95\%CI_{posterior}$[-0.26, 0.31]; easy vs hard: t(43)=0.17, p=.869, d=0.02, 95%CI[-0.27, 0.32], BF_{01}=6.05, $Md_{posterior}$=0.02, $95\%CI_{posterior}$[-0.26, 0.31];

medium vs hard: $t(43)=-0.04$, $p=.972$, $d=-0.01$, 95%CI[-0.31, 0.29], $BF_{01}=6.12$, $Md_{posterior}=-0.01$, $95\%CI_{posterior}$[-0.29, 0.28]).

In Study 3, eleven participants were excluded from the analysis. We did not observe significant effects of ease of encoding ($F(2,112)=2.78$, $p=.067$, $\eta^2=.02$) or group ($F(1,56)=3.27$, $p=.076$, $\eta^2=.01$), and the interaction was also not significant ($F(2,112)=2.64$, $p=.076$, $\eta^2=.01$). However, as in Study 1, ingroup team memory was higher after easily encoded trials compared with medium ($t(56)=2.13$, $p=.038$, $d=0.28$, 95%CI[0.01, 0.55]) and hard trials ($t(56)=2.85$, $p=.006$, $d=0.38$, 95%CI[0.11, 0.65]). There was no significant difference between medium and hard trials ($t(56)=0.44$, $p=.663$, $d=0.06$, 95%CI[-0.20, 0.32]). Outgroup team memory did not significantly differ between ease of encoding levels (easy vs medium: $t(56)=1.20$, $p=.237$, $d=0.16$, 95%CI[-0.11, 0.42], $BF_{01}=3.52$, $Md_{posterior}=0.15$, $95\%CI_{posterior}$[-0.10, 0.40]; easy vs hard: $t(56)=-0.43$, $p=.672$, $d=-0.06$, 95%CI[-0.32, 0.21], $BF_{01}=6.34$, $Md_{posterior}=-0.05$, $95\%CI_{posterior}$[-0.31, 0.20]; medium vs hard: $t(56)=-1.75$, $p=.086$, $d=-0.23$, 95%CI[-0.50, 0.03], $BF_{01}=1.66$, $Md_{posterior}=-0.22$, $95\%CI_{posterior}$[-0.48, 0.04]).

We did not observe significant interactions in Study 1 and 3. However, the pattern of ingroup source memory drop in response to difficult encoding trials was stable. Therefore, we repeated the analysis with the increased power of pooling both samples ($n=101$). We found the expected effect of ease of encoding ($F(2,200)=5.08$, $p=.007$, $\eta^2=.01$) as well as a significant interaction ($F(2,200)=4.34$, $p=.014$, $\eta^2=.01$) between the two factors. The effect of group did not reach significance ($F(1,200)=3.69$, $p=.058$ $\eta^2=.005$). The exploration of the interaction showed the previously described pattern of differences in ingroup team memory between easy and medium ($t(100)=2.93$, $p=.004$, $d=0.29$, 95%CI[0.09, 0.49]) as well as easy and hard trials ($t(100)=4.12$, $p<.001$, $d=0.41$, 95%CI[0.21, 0.61]), but no differences between medium and hard trials ($t(100)=0.91$, $p=.365$, $d=0.09$, 95%CI[-0.11, 0.29]). As before, there were no differences in the outgroup (easy vs medium: $t(100)=1.10$, $p=.276$, $d=0.11$, 95%CI[-0.09, 0.31], $BF_{01}=5.08$, $Md_{posterior}=0.11$, $95\%CI_{posterior}$[-0.09, 0.30]; easy vs hard: $t(100)=-0.29$, $p=.771$, $d=-0.03$, 95%CI[-0.23, 0.17], $BF_{01}=8.71$, $Md_{posterior}=-0.03$, $95\%CI_{posterior}$[-0.22, 0.16]; medium vs hard: $t(100)=-1.49$, $p=.139$, $d=-0.15$, 95%CI[-0.35, 0.05], $BF_{01}=3.11$, $Md_{posterior}=-0.14$, $95\%CI_{posterior}$[-0.34,

0.05]). Furthermore, ingroup and outgroup team memory only differed after hard encoding conditions ($t(100)=-3.20$, $p=.002$, $d=-0.32$, 95%CI[-0.52, -0.12]), but not after easy ($t(100)=1.13$, $p=.262$, $d=0.11$, 95%CI[-0.08, 0.31], $BF_{01}=4.90$, $Md_{posterior}=0.11$, $95\%CI_{posterior}$[-0.08, 0.30]) or medium encoding difficulty ($t(100)=-0.74$, $p=.464$, $d=-0.07$, 95%CI[-0.27, 0.12], $BF_{01}=6.98$, $Md_{posterior}=-0.07$, $95\%CI_{posterior}$[-0.26, 0.12]; see Supplementary Figure 2.1).

The findings therefore corroborate our assumptions and show a reliable effect of ease of encoding on the ingroup, but not the outgroup. While ingroup source memory is responsive to encoding conditions and rather sacrificed when encoding is difficult, outgroup source information is crucial to encode to handle the information appropriately.

Supplementary Figure 2.1

The influence of ease of encoding on team memory in Studies 1 and 3 together

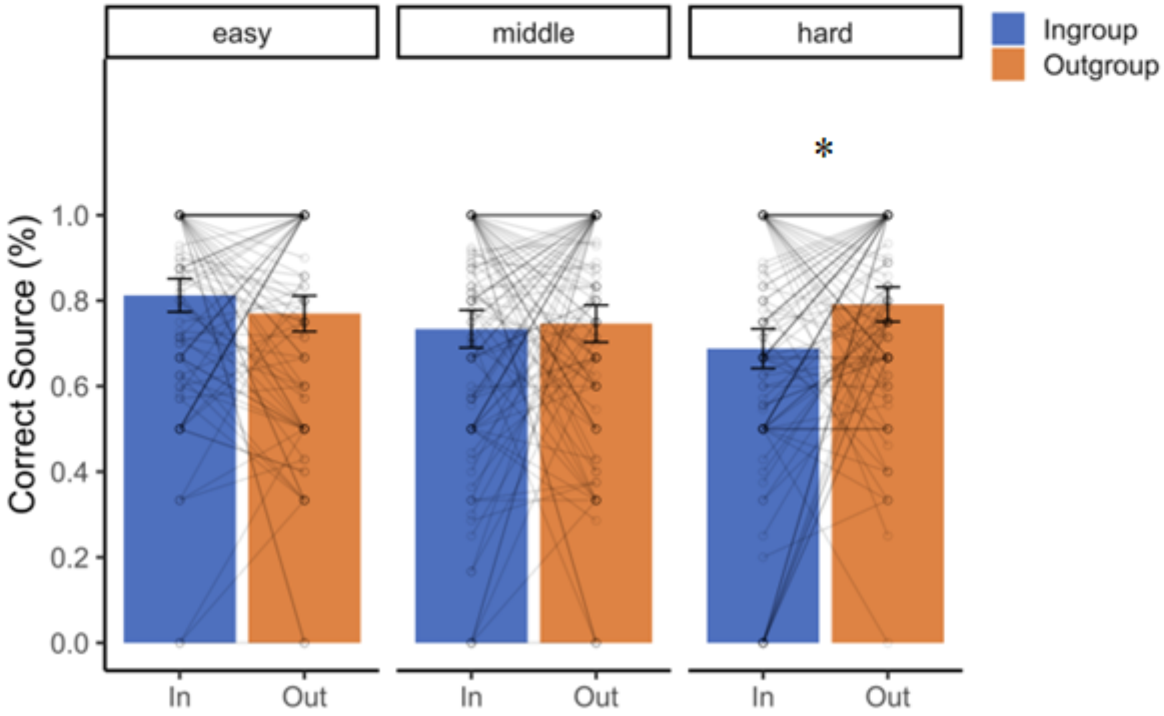

*Note***.** Plotted is the average team memory across the different levels of ease of encoding. The error bars represent standard error of the mean (*SE; n*=101). Each dot represents one participant in a given condition. Highlighted (*) are the comparisons showing a significant group effect (*p*<.05).

Supplementary Note 3: Episodic memory detail is not affected by social group.

Across all studies, we assessed episodic memory detail by asking participants for the position of the object presented by the personas. In the encoding phase, the objects presented by the persona were placed in a unique position on a circle drawn around the face. In Studies 1 and 2, participants were instructed to memorize this position and were tested on it together with source memory (each object was first tested for source and then for position). Study 3 omitted this instruction to align the instructions with prior research [3–5]. Participants were only told that the objects would appear in a random position on the circle. After the source test, a surprise test for those object locations followed.

Detail memory was measured by calculating the Euclidean distance between the object's original position on the screen with the position that participants indicated via mouse click. The experimental program returned coordinates for the mouse clicks in its native screen size units which were used to calculate Euclidian distance to the coordinates of the original object position. To facilitate the interpretation of descriptive detail memory results, a min-max transformation was applied, where the minimal and maximal values in that measure were set to 0 and 1, respectively. Next, the values were inverted so that higher values indicated higher episodic specificity. For the analyses, medians for each participant and condition were calculated due to the high variance in this measure.

In parallel with source memory, we expected a decrease in detail memory after successful inference, but not after unsuccessful inference [3–5]. We also expected better ingroup detail memory, given general ingroup encoding advantages [6,7].

The findings did not support these hypotheses but align with a recent study [8]. Like source memory, we observed higher detail memory after successful inference than unsuccessful inference, probably reflecting better encoding in those trials (see Supplementary Table 3.1). This was also the case in Study 3, where explicit encoding instructions were absent. Furthermore, no group differences and no interactions were found, indicating that detail memory was not affected by the social group manipulation (see Supplementary Table 3.2).

Supplementary Table 3.1

Descriptive Statistics for Detail Memory in Studies 1-3

	Successful Inference		Unsuccessful Inference	
	Ingroup	Outgroup	Ingroup	Outgroup
Study 1	.68 (.19)	.67 (.19)	.62 (.21)	.62 (.20)
Study 2	.66 (.21)	.64 (.19)	.59 (.21)	.59 (.23)
Study 3	.69 (.14)	.67 (.16)	.61 (.19)	.60 (.20)

Note. Group means and standard deviations in parentheses.

Supplementary Table 3.2

ANOVAs for Detail Memory in Studies 1-3

	Inference Success				Social Group				Interaction			
	df	*F*	*p*	η^2	*df*	*F*	*p*	η^2	*df*	*F*	*p*	η^2
1	1,52	7.77	.007	.02	1,52	0.06	.809	0	1,52	0.01	.938	0
2	1,67	11.30	.001	.02	1,67	0.41	.524	.001	1,67	0.43	.512	.001
3	1,67	22.99	<.001	.05	1,67	0.64	.426	.001	1,67	0.24	.626	.001

Supplementary Note 4: Partial source memory is higher in the outgroup.

In Studies 1 and 3, the inclusion of two source personas per group allowed us to test for partial source memory [9] as an additional manipulation check. In this analysis, only incorrect source judgments are included, so trials in which participants did not choose the correct source persona. It is tested whether those errors are preferentially made within a team, so whether a participant who did not choose the correct persona from the ingroup is more likely to wrongly select the other ingroup persona. Partial source memory is calculated as the proportion of errors where a member of the other team is chosen subtracted from the proportion of errors within a team. Scores of zero indicate no preferential errors within a team, scores higher than 0 indicate partial source memory.

In both studies, outgroup partial source memory scores were significantly above zero (Study 1: M=.22, SD=.30; $t(52)$=5.20, p<.001, d=0.71, 95%CI[0.15, 1.28]; Study 3: M=.28, SD=.35; $t(67)$=6.59, p<.001, d=0.80, 95%CI[0.30, 1.30]). Ingroup scores, on the other hand, were not different from zero (Study 1: M=.05, SD=.29, $t(52)$=1.30, p=.199, d=0.18, 95%CI[-0.37, 0.73], BF_{01}=3.01, $Md_{posterior}$=0.17, $95\%CI_{posterior}$[-0.09, 0.43]; Study 3: M=-.01, SD=.31, $t(67)$=-0.23, p=.818, d=-0.03, 95%CI[-0.51, 0.46], BF_{01}=7.32, $Md_{posterior}$=-0.03, $95\%CI_{posterior}$[-0.26, 0.21]). Congruently, outgroup scores were significantly higher than ingroup scores (Study 1: $t(52)$=2.72, p=.009, d=0.37, 95%CI[0.09, 0.65]; Study 3: $t(67)$=4.49, p<.001, d=0.54, 95%CI[0.29, 0.80]).

These results indicate that participants had a higher perception of group for the outgroup. A reduced differentiation of the two personas may have increased partial source memory. Conversely, the ingroup consisted of more individuated members that were less strongly defined by their group identity. This finding of differential individuation is in line with previous research [10,11] and therefore indicates that our manipulation was successful in creating authentic ingroups and outgroups that had similar mnemonic consequences as those demonstrated by previous research.

Supplementary Note 5: Results from all ANOVAs

Supplementary Table 5.1 lists all results from the ANOVAs for the main memory indicators accuracy, response times, and confidence. Supplementary Table 5.2 shows results for source memory.

Supplementary Table 5.1

Results from ANOVAs for Accuracy, Response Times, and Confidence

	Association Type				Social Group				Interaction			
Ind	*df*	*F*	*p*	η^2	*df*	*F*	*p*	η^2	*df*	*F*	*p*	η^2
Study 1												
Acc	2,104	110.33	<.001	.31	1,52	8.16	.006	.01	2,104	2.06	.132	.005
RTs	2,104	305.16	<.001	.52	1,52	1.81	.184	.001	2,104	0.05	.951	0
Con	2,104	72.61	<.001	.18	1,52	11.80	.001	.01	2,104	2.16	.120	.002
Study 2												
Acc	2,134	139.04	<.001	.24	1,67	0.34	.564	0	2,134	1.12	.329	.001
RTs	2,134	206.24	<.001	.41	1,67	3.08	.084	.001	2,134	1.48	.232	.001
Con	2,134	60.47	<.001	.17	1,67	0.28	.600	0	2,134	1.15	.320	.001
Study 3												
Acc	2,134	68.08	<.001	.16	1,67	11.49	.001	.01	2,134	0.10	.905	0
RTs	2,134	151.31	<.001	.40	1,67	0.81	.371	.001	2,134	1.21	.302	.001
Con	2,134	50.47	<.001	.14	1,67	6.14	.016	.004	2,134	3.29	.040	.004

Note. ANOVAs for all studies and Indicators (Ind): Accuracy (Acc), response times (RTs), and confidence ratings (Con). Each ANOVA had the factors association type (AB, BC, AC) and social group (ingroup, outgroup).

Supplementary Table 5.2

Results from ANOVAs for Source Memory

	Inference Success				Social Group				Interaction			
	df	*F*	*p*	η^2	*df*	*F*	*p*	η^2	*df*	*F*	*p*	η^2
1 - P	1,52	10.36	.002	.03	1,52	0	.946	0	1,52	1.07	.305	.002
1 - T	1,52	10.64	.002	.04	1,52	8.90	.004	.03	1,52	6.65	.013	.01
2	1,67	7.90	.006	.02	1,67	0.02	.877	0	1,67	2.41	.125	.004
3 - P	1,67	1.60	.210	.003	1,67	1.11	.295	.003	1,67	0.48	.489	.001
3 - T	1,67	2.98	.089	.01	1,67	4.15	.046	.01	1,67	1.18	.282	.003

Note. The table shows source memory results from all three studies. In Studies 1 and 3, source memory is differentiated into memory for the persona (P) and memory for the team (T).

Supplementary Note 6: Descriptive Values of Direct, Inferential, and Source Memory

The following tables provide all means and standard deviations (in parentheses) for the core analyses. That includes accuracy, response times (RT) and confidence ratings for correct responses in the direct (AB, BC) and inferential (AC) associations (Supplementary Table 6.1) as well as source memory (Supplementary Table 6.2), which is distinguished into source memory for the persona (P) and team (T) when possible.

Supplementary Table 6.1

Descriptive Values of Memory Performance in all studies

	AB		BC		AC	
	Ingroup	Outgroup	Ingroup	Outgroup	Ingroup	Outgroup
Study 1						
Accuracy	.86 (.10)	.83 (.14)	.90 (.10)	.90 (.10)	.73 (.13)	.69 (.15)
RT	1.64 (0.42)	1.67 (0.39)	1.61 (0.37)	1.64 (0.34)	2.54 (0.44)	2.56 (0.52)
Confidence	2.54 (0.32)	2.52 (0.32)	2.66 (0.30)	2.59 (0.34)	2.30 (0.36)	2.22 (0.36)
Study 2						
Accuracy	.86 (.10)	.83 (.14)	.90 (.10)	.90 (.10)	.74 (.12)	.70 (.14)
RT	1.62 (0.41)	1.67 (0.39)	1.60 (0.36)	1.62 (0.33)	2.53 (0.45)	2.57 (0.52)
Confidence	2.54 (0.32)	2.52 (0.32)	2.66 (0.30)	2.59 (0.35)	2.30 (0.36)	2.23 (0.37)
Study 3						
Accuracy	.84 (.14)	.81 (.15)	.89 (.14)	.86 (.16)	.73 (.16)	.69 (.17)
RT	1.69 (0.42)	1.75 (0.41)	1.69 (0.35)	1.68 (0.38)	2.57 (0.67)	2.59 (0.70)
Confidence	2.54 (0.30)	2.52 (0.29)	2.65 (0.32)	2.63 (0.32)	2.37 (0.40)	2.27 (0.38)

Supplementary Table 6.2

Descriptive Values of Source Memory in all studies

	Inference Success		Inference Failure	
	Ingroup	Outgroup	Ingroup	Outgroup
1 – Persona	.56 (.27)	.54 (.22)	.54 (.33)	.49 (.32)
1 – Team	.74 (.17)	.76 (.18)	.67 (.28)	.74 (.28)
2	.76 (.16)	.74 (.19)	.68 (.24)	.71 (.24)
3 – Persona	.57 (.20)	.55 (.19)	.45 (.28)	.48 (.26)
3 – Team	.74 (.15)	.76 (.16)	.61 (.25)	.72 (.23)

Supplementary Note 7. No inference ingroup advantage with one persona per group presenting the BC associations.

In a preliminary experiment (*n*=38), we had employed a similar procedure as Study 2 (so only one source persona per group), but with the difference that the personas presented the BC associations instead of the AB associations. Corroborating the findings from Study 2, we found no effect of group in the AC associations (See Supplementary Table 7.1). However, a group effect was observed in the response times of BCs and the confidence ratings of ABs. Participants were faster at retrieving ingroup BC associations, and they were also more confident retrieving ingroup AB associations in comparison with the respective outgroup associations.

Supplementary Table 7.1

Descriptive values and results of direct contrasts in a preliminary study

	Ingroup		Outgroup		Paired *t*-test				
Type	*M*	*SD*	*M*	*SD*	*df*	*t*	*p*	*d*	95%CI
Accuracies									
AB	.88	.14	.88	.14	37	0	.994	0	[-0.32, 0.32]
BC	.90	.13	.90	.12	37	0.41	.687	0.07	[-0.26, 0.39]
AC	.74	.16	.71	.20	37	1.25	.218	0.20	[-0.12, 0.53]
Response Times									
AB	1.60	0.42	1.60	0.39	37	0.09	.931	0.01	[-0.31, 0.34]
BC	1.67	0.46	1.75	0.46	37	-2.19	.035	-0.35	[-0.69, -0.02]
AC	2.33	0.46	2.37	0.51	37	-0.61	.546	-0.10	[-0.42, 0.23]
Confidences									
AB	2.74	0.24	2.70	0.25	37	2.19	.035	0.36	[0.02, 0.69]
BC	2.73	0.23	2.70	0.28	37	1.14	.263	0.18	[-0.14, 0.51]
AC	2.41	0.43	2.37	0.41	37	1.57	.126	0.25	[-0.07, 0.58]

Note. Means and Standard Deviations in all association types. Ingroup and outgroup values were compared in paired *t*-tests.

Supplementary References

1. Veksler, A. E., & Eden, J. (2017). Measuring interpersonal liking as a cognitive evaluation: Development and validation of the IL-6. *Western Journal of Communication*, *81*(5), 1–16. https://doi.org/10.1080/10570314.2017.1309452

2. JASP Team. (2023). *JASP (Version 0.17.1)*.

3. Carpenter, A. C., & Schacter, D. L. (2017). Flexible retrieval: When true inferences produce false memories. *Journal of Experimental Psychology: Learning, Memory, and Cognition*, *43*(3), 335–349. https://doi.org/10.1037/xlm0000340

4. Carpenter, A. C., & Schacter, D. L. (2018). False memories, false preferences: Flexible retrieval mechanisms supporting successful inference bias novel decisions. *Journal of Experimental Psychology: General*, *147*(7), 988–1004. https://doi.org/10.1037/xge0000391

5. Carpenter, A. C., & Schacter, D. L. (2018). Flexible retrieval mechanisms supporting successful inference produce false memories in younger but not older adults. *Psychology and Aging*, *33*(1), 134–143. https://doi.org/10.1037/pag0000210

6. Jeon, Y. A., Banquer, A. M., Navangul, A. S., & Kim, K. (2021). Social group membership and an incidental ingroup-memory advantage. *Quarterly Journal of Experimental Psychology*, *74*(1), 166–178. https://doi.org/10.1177/1747021820948721

7. Marsh, B. U. (2020). The cost of racial salience on face memory: How the cross-race effect is moderated by racial ambiguity and the race of the perceiver and the perceived. *Journal of Applied Research in Memory and Cognition*, *10*(1), 13–23. https://doi.org/10.1037/h0101790

8. de Araujo Sanchez, M. A., & Zeithamova, D. (2023). Generalization and false memory in acquired equivalence. *Cognition*, *234*, Article 105385. https://doi.org/10.1016/j.cognition.2023.105385

9. Dodson, C. S., Holland, P. W., & Shimamura, A. P. (1998). On the recollection of specific- and partial-source information. *Journal of Experimental Psychology: Learning, Memory, and Cognition*, *24*(5), 1121–1136. https://doi.org/10.1037/0278-7393.24.5.1121

10. Brewer, M. B., Weber, J. G., & Carini, B. (1995). Person memory in intergroup contexts: Categorization versus individuation. *Journal of Personality and Social Psychology*, *69*(1), 29–40. https://doi.org/10.1037/0022-3514.69.1.29

11. Crump, S. A., Hamilton, D. L., Sherman, S. J., Lickel, B., & Thakkar, V. (2010). Group entitativity and similarity: Their differing patterns in perceptions of groups. *European Journal of Social Psychology*, *40*(7), 1212–1230. https://doi.org/10.1002/ejsp.716
