# Supplementary material for: Ingroup sources enhance associative inference
Source: Commun Psychol. 2023 Dec 14;1:40. doi: 10.1038/s44271-023-00043-8 (PMC11332085; doi:10.1038/s44271-023-00043-8)
Supplement: Supplementary file 3 — Reporting Summary [file 44271_2023_43_MOESM3_ESM.pdf]

nature portfolio

Corresponding author(s): Inês Bramão

Last updated by author(s): Nov 11, 2023

Reporting Summary

Nature Portfolio wishes to improve the reproducibility of the work that we publish. This form provides structure for consistency and transparency in reporting. For further information on Nature Portfolio policies, see our Editorial Policies and the Editorial Policy Checklist.

Statistics

For all statistical analyses, confirm that the following items are present in the figure legend, table legend, main text, or Methods section.

n/a	Confirmed	
☐	☒	The exact sample size (*n*) for each experimental group/condition, given as a discrete number and unit of measurement
☐	☒	A statement on whether measurements were taken from distinct samples or whether the same sample was measured repeatedly
☐	☒	The statistical test(s) used AND whether they are one- or two-sided<br>*Only common tests should be described solely by name; describe more complex techniques in the Methods section.*
☐	☒	A description of all covariates tested
☐	☒	A description of any assumptions or corrections, such as tests of normality and adjustment for multiple comparisons
☐	☒	A full description of the statistical parameters including central tendency (e.g. means) or other basic estimates (e.g. regression coefficient) AND variation (e.g. standard deviation) or associated estimates of uncertainty (e.g. confidence intervals)
☐	☒	For null hypothesis testing, the test statistic (e.g. *F*, *t*, *r*) with confidence intervals, effect sizes, degrees of freedom and *P* value noted<br>*Give P values as exact values whenever suitable.*
☐	☒	For Bayesian analysis, information on the choice of priors and Markov chain Monte Carlo settings
☒	☐	For hierarchical and complex designs, identification of the appropriate level for tests and full reporting of outcomes
☐	☒	Estimates of effect sizes (e.g. Cohen's *d*, Pearson's *r*), indicating how they were calculated

Our web collection on statistics for biologists contains articles on many of the points above.

Software and code

Policy information about availability of computer code

Data collection	Data was collected using the survey tool Qualtrics and the experimental program PsychoPy, which was used to conduct the behavioral experiment online via Pavlovia. Participants were recruited via Prolific.
Data analysis	R Studio was used to analyze the data using the packages tidyverse and ez. Plots were made using ggplot2.

For manuscripts utilizing custom algorithms or software that are central to the research but not yet described in published literature, software must be made available to editors and reviewers. We strongly encourage code deposition in a community repository (e.g. GitHub). See the Nature Portfolio guidelines for submitting code & software for further information.

Data

Policy information about availability of data

All manuscripts must include a data availability statement. This statement should provide the following information, where applicable:

- Accession codes, unique identifiers, or web links for publicly available datasets
- A description of any restrictions on data availability
- For clinical datasets or third party data, please ensure that the statement adheres to our policy

All data, aggregated for each participant, is available on OSF (https://osf.io/n3f9p/?view_only=0c97eba9c9264c18afa1d3faefcff01d). All statistical tests can be replicated with these data sets.

Human research participants

Policy information about studies involving human research participants and Sex and Gender in Research.

Reporting on sex and gender	Participants were asked to indicate their gender at the beginning of the survey. They could choose from the options "female", "male", "non-binary / third gender", and "prefer not to say". This was used for sample description only and did not enter analyses as gender differences in the ability to perform associative inference were not expected or the purpose of this study.
Population characteristics	The sample consisted of volunteers from the sampling provider Prolific. They were between 20-34 years old (overall M=25.8) and 88 were female, 94 were male, and 7 were non-binary / third gender. No demographic characteristics entered tbe analyses. Ethnicity was not assessed.
Recruitment	Prolific offers a large pool of participants that can take part in studies for compensation. Once published eligible participants can decide to take part in the study and also end participation at any point. Within this convenience sampling method, there is a bias for motivated participants that aim to make money through participation in experiments. Only US-American participants were eligible to participate and due to decline of memory capacity, participants had to be <35 years old. All participants provided informed consent and were compensated financially according to prolific recommendations.
Ethics oversight	All methods were conducted in accordance with the Swedish Act concerning the Ethical Review of Research involving Humans (2003:460) and the Code of Ethics of the World Medical Association (Declaration of Helsinki). As established by Swedish authorities and specified in the Swedish Act concerning the Ethical Review of Research involving Humans (2003:460), the present study does not require specific ethical review by the Swedish Ethical Review Authority due to the following reasons: (1) it does not deal with sensitive personal data, (2) it does not use methods that involve a physical intervention, (3) it does not use methods that pose a risk of mental or physical harm, (4) it does not study biological material taken from a living or dead human that can be traced back to that person. Given the compliance with these laws and rules, we did not seek local ethics committee approval in the United States, where participants were based, either.

Note that full information on the approval of the study protocol must also be provided in the manuscript.

Field-specific reporting

Please select the one below that is the best fit for your research. If you are not sure, read the appropriate sections before making your selection.

☐ Life sciences ☒ Behavioural & social sciences ☐ Ecological, evolutionary & environmental sciences

For a reference copy of the document with all sections, see nature.com/documents/nr-reporting-summary-flat.pdf

Behavioural & social sciences study design

All studies must disclose on these points even when the disclosure is negative.

Study description	The research was a purely quantitative behavioral experiment.
Research sample	The sample consisted of volunteers from the sampling provider Prolific from the United States. They were between 20-34 years old (overall M=25.8) and 88 were female, 94 were male, and 7 were non-binary / third gender. The sample was not representative but depended on voluntary participation. The age limits were set due to declining memory ability during aging and US-Americans were chosen as the sample because they represent the biggest group on Prolific, the experiment could be conducted in English with them, and the US-American political system makes a clear distinction between the two major parties, which was assumed to aid our group manipulation. All participants provided informed consent and no deception or blinding was necessary for the experiment. They were also informed how to leave the study and that their data would be deleted if they did so. Participants were fully debriefed after the experiment and received financial compensation in accordance with prolific guidelines.
Sampling strategy	A convenience sample of Prolific workers was obtained. Minimal sample size for Study 1 was determined based on previous literature. The effect from this study informed the sample sizes of Study 2 and 3, which was determined using a bootstrapping method in which n participants were randomly selected from the sample of Study 1 and used to repeat the analysis. For each sample size n, this was repeated 1,000 times. It was found that with n = 68, the effect from Study 1 was reliably detected (power = 80%).
Data collection	Data was collected online using Qualtrics, PsychoPy, and Prolific. No experimenter was present at testing and the experiment was fully self-conducted. Therefore, no blinding was necessary during data collection.
Timing	Study 1: October 2021 to February 2022; Study 2: August 2022 to October 2022; Study 3: October 2022 to November 2022
Data exclusions	The exclusion criteria were pre-established and in the cases of Study 2 and 3 also pre-registered. In Study 1, seven participants were excluded because they did not produce incorrect inference trials, which made the source memory analysis impossible. In Study 2, nine participants were excluded for that reason, six participants were excluded because they did not answer to the ease of encoding questions 90% of the time and thus may not have been attentive during encoding, and two were excluded due to a failed group

	manipulation. In Study 3, compliance with the latter two criteria was monitored online and participants were screened out directly upon failure. In addition to that, one participant was excluded because they did not choose the correct personas as ingroup and outgroup after the experiment and may therefore not have recognized them during the task. Furthermore, six participants were excluded for not providing incorrect inference trials.
Non-participation	As data collection was online, it is impossible to say how many participants stopped the task during the session or decided not to participate after reading the information or instructions. While participants can "return" their submission, meaning that they changed their mind and decided not to take part, we cannot determine whether that was after finding out more about the task or before any familiarization with the experiment due to the anticipated length of the experiment or a change of plans. However, no participant refused data usage after the experiment.
Randomization	Experimental manipulation was within-participant, so each participant had trials in each condition. The assignment of the encoding material to the conditions was counter-balanced, as were the encoding and test orders.

Reporting for specific materials, systems and methods

We require information from authors about some types of materials, experimental systems and methods used in many studies. Here, indicate whether each material, system or method listed is relevant to your study. If you are not sure if a list item applies to your research, read the appropriate section before selecting a response.

Materials & experimental systems

n/a	Involved in the study
☒	☐ Antibodies
☒	☐ Eukaryotic cell lines
☒	☐ Palaeontology and archaeology
☒	☐ Animals and other organisms
☒	☐ Clinical data
☒	☐ Dual use research of concern

Methods

n/a	Involved in the study
☒	☐ ChIP-seq
☒	☐ Flow cytometry
☒	☐ MRI-based neuroimaging
